# Post-COVID-19 pneumonia lung fibrosis: a worrisome sequelae in surviving patients

**DOI:** 10.1186/s43055-021-00484-3

**Published:** 2021-04-13

**Authors:** Rasha Mostafa Mohamed Ali, Mai Bahgat Ibrahim Ghonimy

**Affiliations:** Diagnostic & Interventional Radiology Department, Gastrointestinal Tract and Thoracic Imaging Units, Kasr Al-Aini Hospitals, Cairo University, Cairo, Egypt

**Keywords:** Post-COVID-19, Computed tomography, Pulmonary fibrosis, Worrisome complication

## Abstract

**Background:**

Progressive fibrotic lung disease is one of the possible consequences of COVID-19 pulmonary pneumonia, and it is one of the most worrying long-term complications. Pulmonary fibrosis is associated with non-reversible lung dysfunction. The long-term lung changes of previous COVID-19 infection still not completely understood and should be included in further studies. The aim of this study is the early detection and prediction of patients whom may develop such serious complication, thus giving a chance for early introduction of anti-fibrotic drugs.

**Results:**

From April 2020 to December 2020, 80 patients in Cairo, Egypt, who have clinical manifestations and confirmed COVID-19 by PCR, were evaluated by follow-up MDCT. CT image analysis was processed including comparative study using follow-up data (different radiological signs and residual fibrotic changes). Although there was no specific cause for post-COVID-19 lung fibrosis, there were some predicting factors such as old age, cigarette smoking, high CT severity score, and long-term mechanical ventilation.

**Conclusion:**

Early detection of potential cases of post-COVID-19 pulmonary fibrosis may give a chance to prevent or at least modify such disabling complication.

## Background

By the end of November 2020, over 67 million people had been confirmed infected all over the world, with over 1.5 million deaths. The symptoms associated with COVID-19 are diverse, ranging from mild upper respiratory tract symptoms to severe acute respiratory distress syndrome [1].

The primary risk factors for severe COVID-19 are aging, male sex, and comorbidities such as diabetes and hypertension [2].

Following the COVID-19 outbreak, there will be raising number of patients worldwide who have survived COVID-19 but still suffer symptoms, even after they have been laboratory tested negative for the disease, thus, raising the importance of managing this COVID-19 sequelae. COVID-19 sequelae may range from mild form of fatigue to serious forms requiring long-term oxygen therapy or even lung transplantation owing to pulmonary fibrosis [3].

Preceding coronavirus epidemics have been accompanied with considerable post-viral fibrosis and physical disabilities. Frequent follow-up of patients after COVID-19 is mandatory [4]. COVID-19 leads to a broad variety of respiratory diseases with high occurrence of acute respiratory distress syndrome [5].

The load of fibrotic pulmonary changes following SARS-CoV-2 infection is likely to be high; thus, the global burden of fibrotic pulmonary disease will be increasing significantly [6]. Fibrosis is not common following other viral pneumonias and has almost never been reported after H1N1 pneumonia. Fibrotic changes have been reported, however, in about 8% of patients with SARS and 20% of patients with H7N9 influenza [7].

About 92 million people have already been affected by COVID-19 in the course of this pandemic. While the majority have mild form of infection, about 15% will get a severe COVID-19 pneumonia, and 5% will progress to ARDS, meaning that almost 4.8 million will have sever pulmonary involvement. In spite most of them will cure without lung damage, most likely a significant number of patients will suffer residual sequelae [8]. Although till now, there is not a completely proven treatment of post-COVID 19 pulmonary fibrosis; the use of anti-fibrotics in the early acute phase of severe disease with ARDS may reduce fibrosis [9].

The aim of this study is the early detection and prediction of patients who may develop such serious complication, and thus giving a chance for early introduction of anti-fibrotic drugs.

## Methods

### Study design

In this cross-sectional prospective study, 80 patients including 40 males (50%) and 40 females (50%) with age range from 25 to 75 years old (mean age of 43.2 years old) were enrolled in this study in Cairo, Egypt, during the period from 10 April 2020 to 30 December 2020. The male-to-female ratio was designed to 1:1. Patients with age range 25–45 years old were 25 patients, from 45 to 60 years old were 25 patients, and those ranging from 60 to 75 years old were 30 patients. All patients were subjected to full clinical data taking including age, sex, exposure history, and clinical complaint.

### Inclusion criteria

For patients having positive CT chest findings of COVID-19 with confirmed diagnosis by polymerase chain reaction test, follow-up CT chest was done following negative PCR result to assess degree of recovery and residual fibrotic changes. Further follow-up at 4–6 weeks and 9–12 weeks for patients with residual symptoms and/or residual lung fibrotic changes was done.

### Exclusion criteria

Pregnant females, patients with severe respiratory motion artifacts on CT images, patients with history of chronic interstitial lung disease, and patients with any chronic medical condition such as DM, hypertension, and autoimmune disease.

### Method

All patients underwent non-contrast-enhanced chest CT using multi-detector CT (MDCT) scanner with 64 channels. The detailed parameters for CT acquisition were as discussed in Table 1 using sharp reconstruction kernel. CT images were obtained with the patient in the supine position at full inspiration, foot first, and without contrast medium (Table 1).

**Table 1:** Parameters for CT acquisition

Tube voltage	120 kVp
Tube current	Standard (reference mAs, 60–120) Low-dose (reference mAs, 30) with automatic exposure control
Slice thickness	1.0 mm
Reconstruction interval	1.0–3.0 mm

All images were assessed in both lung window of 1000 WW and – 600 WL and mediastinal window of 400 WW and 60 WL using post-acquisition 2D coronal and sagittal images reformatting for better assessment of the disease distribution.

Different CT chest finding like ground glass opacities, consolidation, vascular thickening, and bronchial thickening were assessed regarding their location and distribution, whether unilateral or bilateral; peripheral, central, and lobe predominance. CT imaging features such as bronchovascular bundle distortion, fibrotic strips, traction bronchiectasis, architectural distortion, and interlobar septal thickening are suggestive of pulmonary fibrosis. CT was done after initial diagnosis and after having negative PCR results at 4–6 weeks interval as well as at 9–12 weeks. Two radiologists with 15 years of experience in thoracic imaging, while being blinded to the clinical data, independently assessed the CT image analysis; as well as a CT severity score in each study.

### CT severity score

It is a score for degree of lung affection based on dividing the lung into five lung lobes; each lobe affection was visually scored on a scale of 0–5, with 0 indicating no involvement, 1 indicating less than 5% involvement, 2 indicating 5–25% involvement, 3 indicating 26–49% involvement, 4 indicating 50–75% involvement, and 5 indicating more than 75% involvement. The total CT score was the sum of the individual lobar scores and ranged from 0 (no involvement) to 25 (maximum involvement) [10, 11].

### Ethical consideration

All patients provided written informed consent. The results of this research were used only in scientific purposes and not in any other aims, and the confidentiality of were completely protected.

### Statistical analysis

Findings are presented as medians and interquartile ranges due to small sample size, and categorical variables are described as whole numbers with percentages in brackets.

## Results

**Fig. 1:**
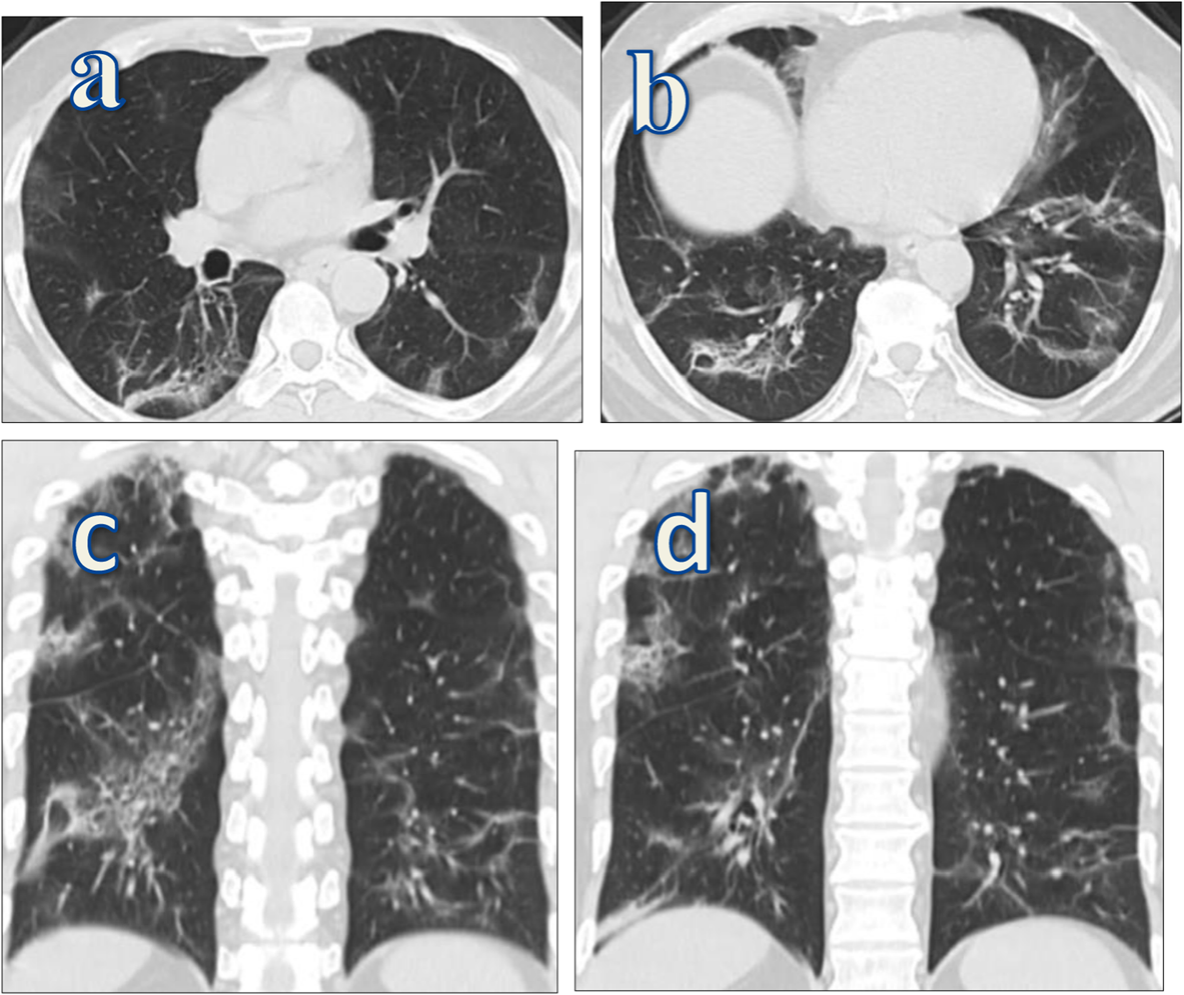
A 56-year-old cigarette smoker male patient presented with ferver, dyspnea, and dry cough diagnosed as positive for COVID-19 by PCR. CT-SS on admission was 16/25. Follow-up axial (**a**,**b**) and coronal (**c**,**d**) MSCT chest was done after 6 weeks from start of symptoms revealed bilateral pulmonary fibrotic changes in the form of fibrotic bands, peribronchial thickening, traction bronchiactasis. and bronchioloctasis

**Fig. 2:**
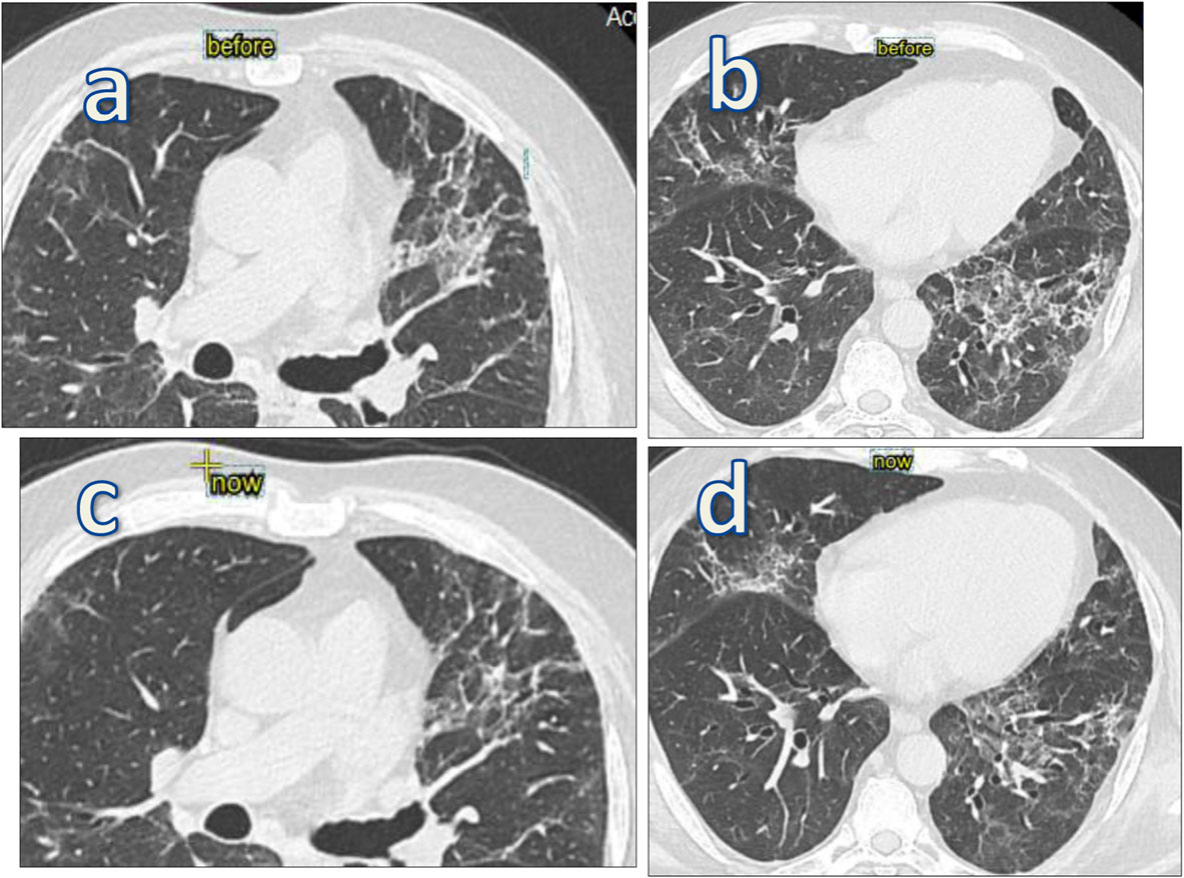
A 64-year-old male patient presented with fever, severe dysnea, and cough; first MSCT was done on the 12th day of manifestation showing classic CT finding of COVID-19, CT-SS 18/25, (bilateral peripheral subpleural ground glass opacifiection with peribronchial thickening and atelactatic bands) (**a**, **b**). Patient came on 12th week with persistent dysnea, axial cuts MSCT (**c**, **d**) was done showing regression regarding ground glass opacities, with persistent fibrotic changes (peribronchial fibrosis, archeticture distortion, and traction bronchectatic and bronchilectatic changes)

This prospective cross section study included 80 patients (40 males, 40 females) with age ranging from 25 to 75 years (mean age of 43.2 years), with confirmed diagnosis by PCR-positive COVID results. They were referred to perform MSCT of the chest as diagnostic and follow-up method. CT was performed at initial presentation then follow-up CT chest was done following negative PCR result to assess degree of recovery and residual fibrotic changes. Further follow-up at 4–6 weeks and 9–12 weeks for patients with residual symptoms and/or residual lung fibrotic changes was done (Figs. 1 and 2).

Most patients presented with dry cough, which was seen in 60 patients (75%); 45 patients suffered from dyspnea (56 %), 40 patients had fever (50%), and 25 patients presented with diarrhea (31%) (Table 2).

**Table 2:** Clinical history of patients enrolled in our study

Number of patients	Clinical history
60 (75%)	Dry cough
45 (56%)	Dyspnea
40 (50%)	Fever
25 (31%)	Diarrhea

*NB* some patients had more than one clinical history

Patients who gave history of heavy cigarettes smoking were 30 patients (37.5%). (More than 20 cigarettes/day for more than 10 years.)

**Fig. 3:**
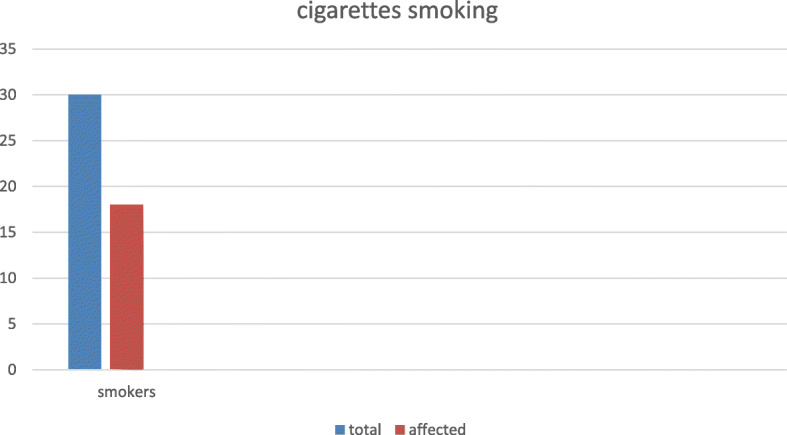
Cigarettes smoker showed much higher incidence of post pulmonary fibrosis than nonsmoking one

Post-COVID-19 pulmonary fibrosis was highly correlated to patient ranging from 60 to 75-year age group (13/30 patients; 43.3%) followed by mild higher prevalence in 45–60-year age group (7/25 patients; 28%), than 25–45-year age group (5/25 patients; 20%). Patient with history of cigarettes smoking showed much higher incidence of post-pulmonary fibrosis than non-smoking one. As from the 30 smoking patients, 18 developed post-pulmonary fibrosis (60%) (Fig. 3).

**Fig. 4:**
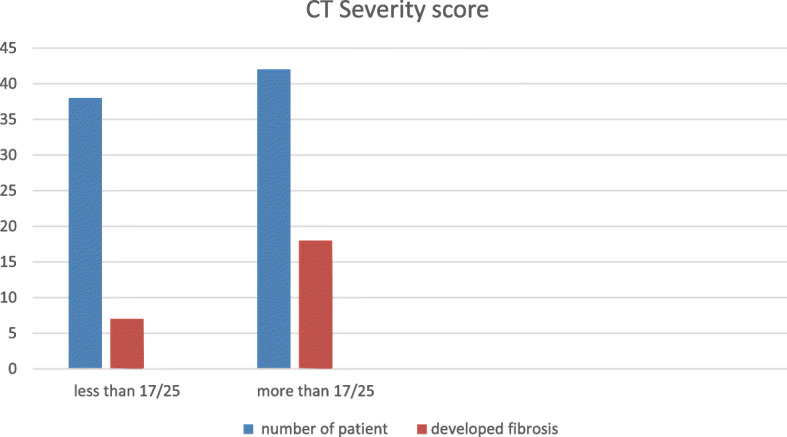
CT severity score above 17/25 showed much higher incidence of post-COVID-19 pulmonary fibrosis

The mild group (CT-SS of 1–17) (38 patients) showed less liability for post-COVID-19 fibrosis seen only in 7 patients (18.4%) whereas the severe group (CT-SS of 18–25) (42 patients) showed higher incidence of post-COVID-19 pulmonary fibrosis seen in 18 patients (42.8%) (Fig. 4).

**Fig. 5:**
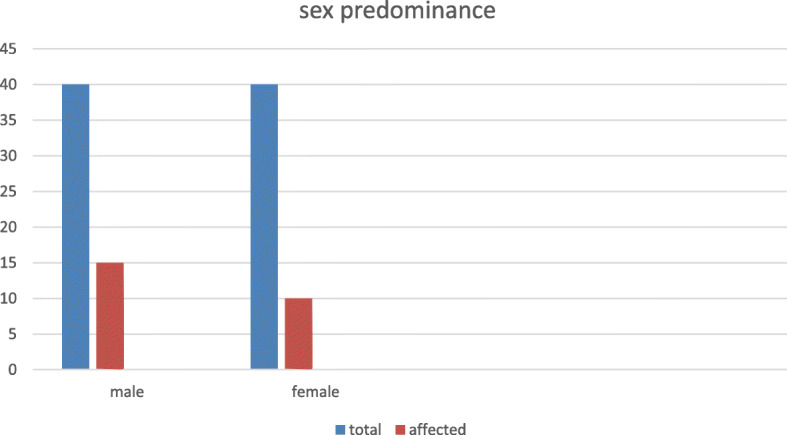
Males are 1.3 times more subjected to post-COVID-19 pulmonary fibrosis than females

**Fig. 6:**
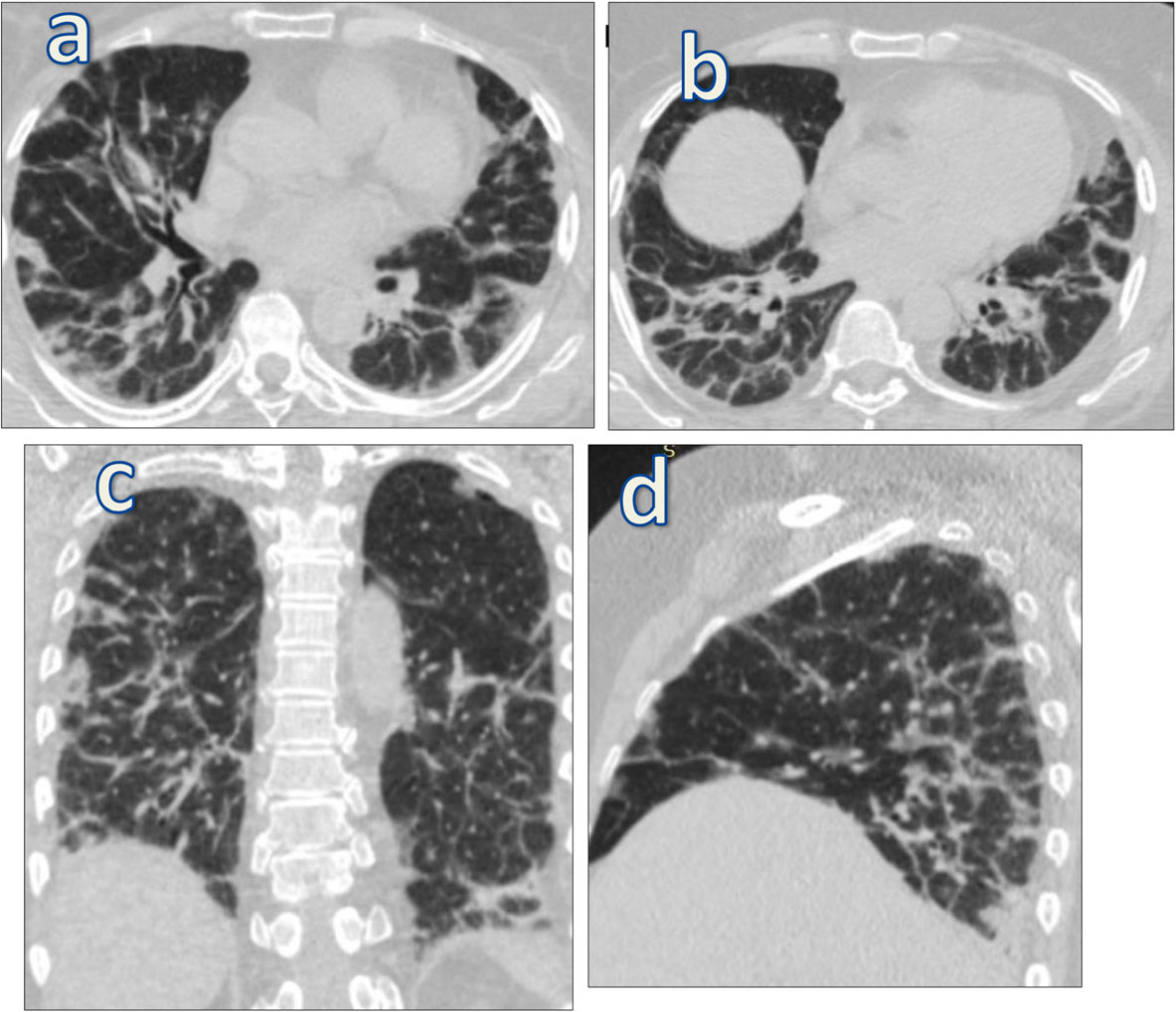
A 60-year-old male patient presented to emergancy room by severe dyspnea; SpO2 was 79%. PCR was positive for COVID-19 and initial CT chest revealed CT-SS 19/25. He was admitted to ICU for 3 weeks. Follow-up MDCT chest axial (**a**,**b**), coronal (**c**), and sagittal (**d**) reconstruction was done 5 weeks later due to persistent hypoxia (SpO2 94%) and showed extensive pulmonary architecture distortion, thick fobrotic bands, and traction bronchiactatic changes denoting post-COVID-19 pulmonary fibrosis. He was discharged on oxygen home therapy, corticosteroid, and anti-fibrotic theray was given

The current study revealed that male showed more susceptibility of having post-COVID-19 pulmonary fibrosis than females, as 15 males out of total 40 males proceeded to post-COVID-19 fibrosis (37.5%) in comparison to female patients only 10 patients complicated with post-COVID-19 lung fibrosis (25%), thus males are around 1.5 times more subjected to post-COVID-19 pulmonary fibrosis than females (Figs. 5 and 6).

**Fig. 7:**
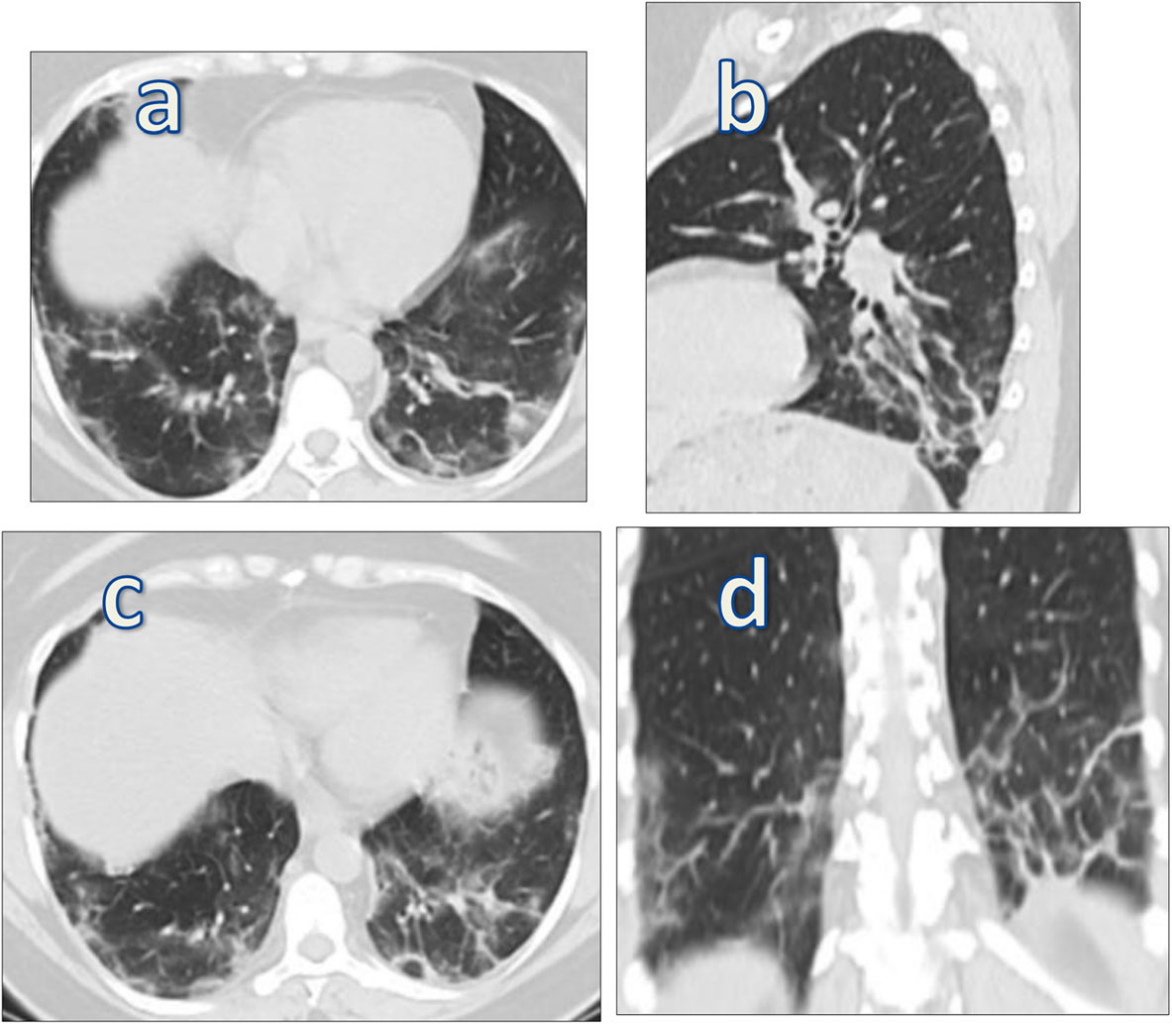
A 51-year-old female patient presented with dyspnea and dry cough, proved to be COVID-19 (positive by PCR). CT-SS on admision was 12/25. Follow-up MDCT chest (axial, sagittal, and coronal reconstructive images) after 4 weeks (**a**, **b**) revealed bilateral lower lobar fibrotic changes with thick parenchymal bands, subpleural lines, and traction bronchiactasis and bronchiolectasis, which became more extensive in follow-up films at 12 weeks (**c**, **d**)

**Fig. 8:**
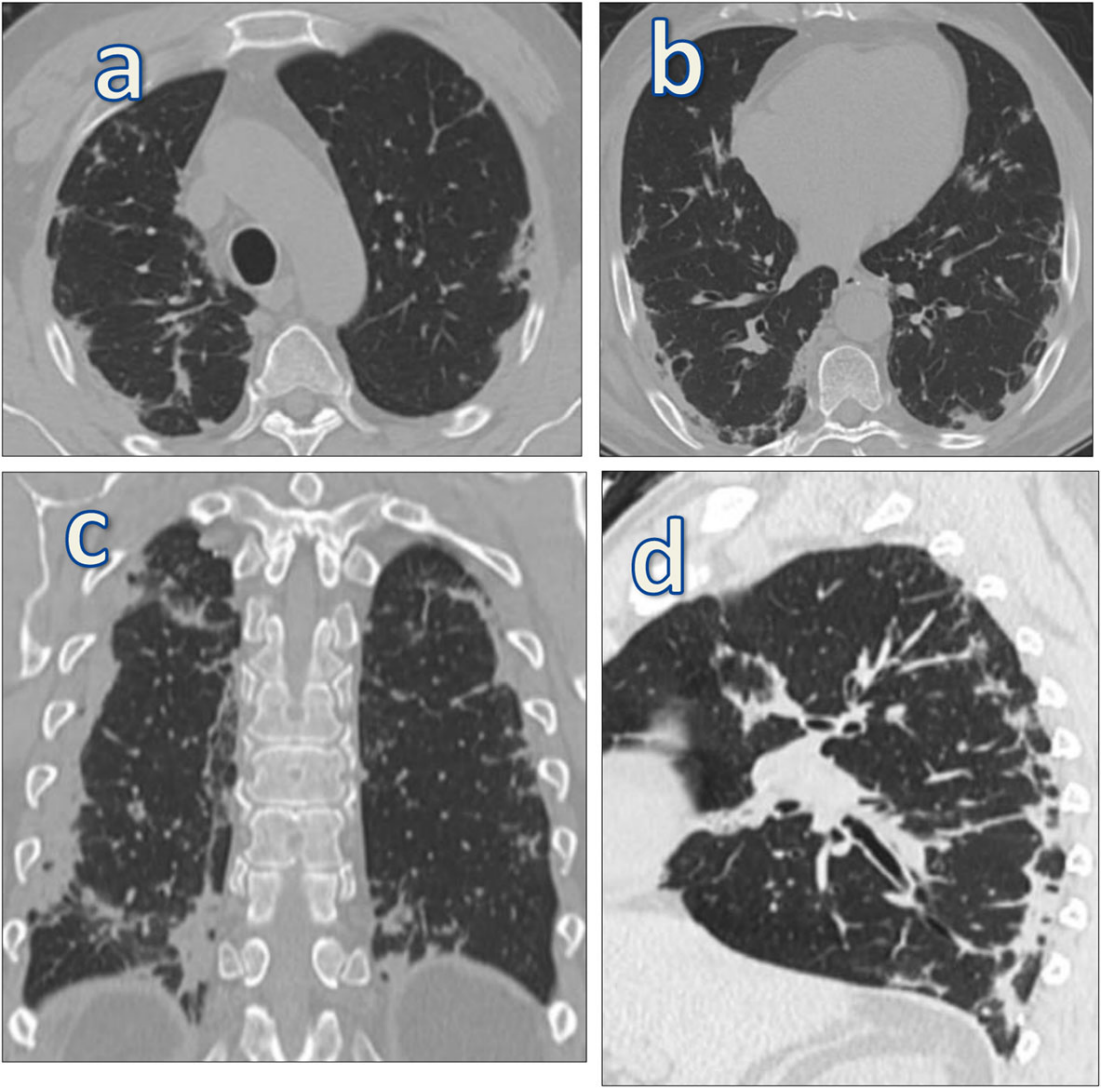
A 45-year-old male patient presented with fever, dry cough, and dyspnea. Initial MSCT was done and revealed bilateral multi-focal subpleural patchy areas of ground glass opcities scattered in both lung fields with CT-SS 8/25. Follow-up MSCT chest after 6 weeks (axial, coronal, and sagittal images) (**a**–**d**) revealed bilateral subpleural interstirial thickening with fibrotic bands and mild traction bronchiactatitic changes, which showed almost silimar findings on 10 weeks follow-up

CT imaging features suggesting fibrosis were bronchovascular bundle distortion, fibrotic strips, traction bronchiectasis, architectural distortion, subplerual curvilinear atelectasis, and interlobular septal thickening (Figs. 7 and 8).

## Discussion

The clinical manifestations of corona virus disease-2019 (COVID-19) can range from mild symptoms to severe illness that lead to permanent lung damage or even mortality [12]. Most of mild and moderate cases have completely recovered but only small proportion of severe cases with acute respiratory distress syndrome (ARDS) continued to remain hypoxemic despite receiving adequate medical treatment [12]*.*

Post-COVID pulmonary fibrosis has been recognized as a potential worrying sequela among survivors as they develop permanent pulmonary architectural distortion and irreversible pulmonary dysfunction [13].

Many theories were discussed as a potential cause of post-COVID pulmonary fibrosis; one of them is cytokine storm which is caused by an abnormal immune mechanism that leads to initiation of pulmonary fibrosis. The reason remains unknown why certain individuals recover from such an insult, while others develop progressive pulmonary fibrosis [14].

In this study, CT chest abnormalities were recorded initially 3–4 weeks after onset of clinical manifestations as well as 10–12 weeks later for monitoring development and/or progression of post-COVID-19 pulmonary fibrosis. In this research, many risk factors were correlated to predict possibility of development of post-COVID-19 pulmonary fibrosis such as advanced age, sex, cigarette smoking, prolonged ICU admission, and CT severity score ( CT-SS).

It was found that post-COVID-19 pulmonary fibrosis was highly correlated to patient age as (13 patients out of 30; 43.3%) who developed pulmonary fibrosis had age ranging from 60 to 75-year age group. This is matching to study by Wong et al. [15], who stated that older people are more likely to develop pulmonary fibrosis following MERS. Low incidence was noted in 45–60-year age group (7 patients out of 25; 28%), and 25–45-year age group showed least incidence (5 out of 25 patients; 20 %); this was also noticed by Das K.M, et al. [16] that correlated age with MERS and SARS-CoV 2 pulmonary fibrosis development.

This current study revealed that males are 1.3 times more subjected to post-COVID-19 pulmonary fibrosis than females, as 15 males out of total of 40 males proceeded to post-COVID-19 fibrosis (37.5%) in comparison to female patients with only 10 patients complicated with post-COVID-19 lung fibrosis (25%). This may be explained by the effect of androgen which promotes the transcription of transmembrane protease, serine 2 gene. That encoded protein primes the spike protein of SARS-Cov-2, thus impair antibody response and facilitate fusion of the virus and host cells [17].

Another risk factor was cigarette smoking; this study showed that cigarette smoker had much higher incidence of post-pulmonary fibrosis than non-smoking one. As from the 30 smoking patients, 18 developed post-pulmonary fibrosis (60%). That was stated by Vardavas C.I., et al. [18] that smokers are 1.4 times more likely to have severe symptoms of COVID-19 and 2.4 times more likely to need ICU admission and mechanical ventilation or die compared to non-smokers patients.

CT severity score (CT-SS) also plays an important role in prediction of disease progression; in this study, we found that the mild group (CT-SS of 1–17) (38 patients) showed less liability for post-COVID-19 fibrosis that developed only in 7 patients (18.4%) whereas the severe group (CT-SS of 18–25) (42 patients) showed higher incidence of post-COVID-19 pulmonary fibrosis seen in 18 patients (42.8%). That is matching with the study of Zhou F., et al. [19] who stated that increased disease severity is a reliable indicator of lung tissue destruction and correlates with mortality risk. According to the World Health Organization (WHO), 80% of SARS-CoV-2 infections are mild, 14% develop severe symptoms, and 6% will become critically ill.

The role of anti-fibrotic drugs in prevention and treatment of post-COVID pulmonary fibrosis is not yet clear at present time. However, these drugs are believed to be useful in patients with acute exacerbations of ILD (both IPF and other fibrotic ILDs), thus reduces pulmonary damage and regressing morbidity and mortality rates in high-risk individuals [14].

This study faced some limitations such as limited number of patients, exclusion of high-risk group of comorbidities such as diabetes and hypertension as well as short-term follow-up that caused lack of correlation with treatment protocols.

## Conclusion

Post-COVID-19 pulmonary fibrosis is one of the most worrying pulmonary complications as it causes permanent lung damage, so prediction of potential high-risk patients may help in applying early medical treatment strategies such as anti-fibrotic drugs, thus reducing disease morbidity and mortality rates.

## Data Availability

Data are available within the article or its supplementary materials.

## Abbreviations

2019-nCOV: 2019 novel coronavirus
ARDS: Acute respiratory distress syndrome
COVID-19: Corona virus disease
CT: Computed tomography
CT-SS: Computed tomography severity score
H9N7: Influenza A virus subtype H9N7
MDCT: Multi-detector computed tomography
MSCT: Multi-slice computed tomography
PCR: Polymerase chain reaction
RT-PCR: Reverse transcriptase polymerase chain reaction
SARS-Cov-2: Severe acute respiratory syndrome coronavirus 2
WHO: World Health Organization

## References

[1] Peter M, Athol U, Jenkins RG (2020). Pulmonary fibrosis and COVID-19: the potential role for antifibrotic therapy. Lancet.

[2] George P, Patterson C, Ak R (2019). Lung transplantation for idiopathic pulmonary fibrosis. Lancet Respir Med.

[3] Carfì A, Bernabei R, Landi F (2020). For the Gemelli against COVID-19 post-acute care study group. Persistent symptoms in patients after acute COVID-19. J Am Med Assoc.

[4] King CS, Nathan SD (2017). Idiopathic pulmonary fibrosis: effects and optimal management of comorbidities. Lancet Respir Med.

[5] Richeldi L, du Bois R, Raghu G (2014). Efficacy and safety of nintedanib in idiopathic pulmonary fibrosis. N Engl J Med.

[6] Jr King, Bradford WZ, Castro-Bernardini S (2014). A phase 3 trial of pirfenidone in patients with idiopathic pulmonary fibrosis. N Engl J Med.

[7] Zarir F, Priyanka K, Awatansh K (2021). Fibrotic interstitial lung disease occurring as sequelae of COVID-19 pneumonia despite concomitant steroids. Lung India.

[8] Carsana L, Sonzogni A, Nasr A (2020). Pulmonary post-mortem findings in a series of COVID-19 cases from northern Italy: A two-center descriptive study. Lancet Infect Dis.

[9] Wu C, Chen X, Yanping C (2020). Risk factors associated with acute respiratory distress syndrome and death in patients with coronavirus disease 2019 pneumonia in Wuhan. China JAMA Intern Med.

[10] Francone M, Iafrate F, Gorgio M (2020). Chest CT score in COVID-19 patients: correlation with disease severity and short-term prognosis. Eur Radiol.

[11] Pan F, Ye T, Sun P, Gui S, Liang B, Li L, Zheng D, Wang J, Hesketh RL, Yang L, Zheng C (2020). Time course of lung changes at chest CT during recovery from coronavirus disease 2019 (COVID-19). Radiology.

[12] Tale S, Ghosh S, Meitei SP (2020). Post-COVID-19 pneumonia pulmonary fibrosis. Int J Med.

[13] Ademola S, Simon A, Oyeronke T (2020). Pulmonary fibrosis in COVID-19 survivors: predictive factors and risk reduction strategies. Pulmon Med.

[14] George PM, Wells AU, Jenkin RG (2020). Pulmonary fibrosis and COVID-19: the potential role for antifibrotic therapy. Lancet Respir Med.

[15] Wong K, Antonio GA, Hui DS (2002). Severe acute respiratory syndrome: thin-section computed tomography features, temporal changes, and clinicoradiologic correlation during the convalescent period. J Comput Assisted Tomogr.

[16] Sansone A, Mollaioli D, Ciocca G, Limoncin E, Colonnello E, Vena W, Jannini EA (2021). Addressing male sexual and reproductive health in the wake of COVID-19 outbreak. J Endocrinol Invest..

[17] Lee EY, Singh R (2017). Follow-up chest radiographic findings in patients with MERS-CoV after recovery. Indian J Radiol Imag.

[18] Vardavas CI, Nikitara K (2020). COVID-19 and smoking: a systematic review of the evidence. Tob Induc Dis..

[19] Zhou F, Yu T, Du R (2020). Clinical course and risk factors for mortality of adult inpatients with COVID-19 in Wuhan, China: a retrospective cohort study. Lancet.

